# Trimethylamine-N-Oxide Promotes Osteoclast Differentiation and Bone Loss via Activating ROS-Dependent NF-κB Signaling Pathway

**DOI:** 10.3390/nu14193955

**Published:** 2022-09-23

**Authors:** Ning Wang, Yongqiang Hao, Lingjie Fu

**Affiliations:** Shanghai Key Laboratory of Orthopaedic Implants, Department of Orthopaedic Surgery, Shanghai Ninth People’s Hospital, Shanghai Jiao Tong University School of Medicine, Shanghai 200011, China

**Keywords:** trimethylamine-N-oxide, osteoclast, NF-κB signaling pathway, reactive oxygen species, osteoporosis

## Abstract

Trimethylamine-N-oxide (TMAO), an important gut microbiota (GM)-derived metabolite, has been shown to be abnormally increased in osteoporosis. However, the role and underlying mechanism of TMAO in regulating bone loss during osteoporosis have not been fully investigated. In the current study, we found that 100–400 μM TMAO dose-dependently enhanced TRAP-positive osteoclasts, F-actin ring formation, and resorption area on bovine bone slices and up-regulated osteoclast-related gene expression (Calcr, Traf6, Dcstamp, Acp5, C-Fos, and NFATc1). Western blotting validated that TMAO not only activated NF-κB signaling pathway but also stimulated c-Fos and NFATc1 protein expression in a dose-dependent manner. Furthermore, BAY 11-7082, an NF-κB inhibitor, pretreatment markedly suppressed TRAP-positive osteoclast formation and osteoclast-related genes under TMAO treatment. BAY 11-7082 also inhibited p-p65/p65, c-Fos, and NFATc1 protein expression promoted by TMAO. Moreover, TMAO significantly increased ROS production, which was inhibited by N-acetylcysteine (NAC), an ROS antagonist. In addition, we proved that NAC pretreatment could inhibit TMAO-promoted NF-κB activation. NAC also suppressed TRAP-positive osteoclast formation, osteoclast-related gene expression, and protein expression of c-Fos and NFATc1 under TMAO treatment. In vivo studies showed significantly decreased bone mass and increased TRAP-positive osteoclasts in TMAO-treated C57BL/6 mice. Moreover, western-blotting and immunohistochemical staining showed that TMAO administration markedly stimulated NF-κB p65 expression. Additionally, TMAO administration significantly promoted the gene and protein expression of C-Fos and NFATc1. In conclusion, TMAO could promote osteoclast differentiation and induce bone loss in mice by activating the ROS-dependent NF-κB signaling pathway.

## 1. Introduction

Trimethylamine-N-oxide (TMAO), a critical gut microbiota (GM)-derived metabolite, is produced from dietary L-carnitine and phosphatidylcholine-rich foods such as red meat, dairy, and eggs [1]. These dietary nutrients are digested by GM to produce trimethylamine (TMA) before being transported to the liver, where TMA is converted to TMAO by flavin monooxygenase 3 (FMO3) [2]. Elevated plasma TMAO results in increased pro-inflammatory cytokines and eventually contributes to several inflammation-related diseases, including cardiovascular disease, diabetes, stroke, and metabolic syndrome [3,4,5].

Osteoporosis is mainly induced by increased levels of inflammatory factors, such as nuclear factor-kappaB ligand (RANKL) and tumor necrosis factor-alpha (TNF-alpha) [6]. At present, it is accepted that osteoporosis is an inflammation-related disease. A recent study showed that aged mice with low bone mass had increased inflammatory factors and serum TMAO levels [7]. Moreover, TMAO was correlated with bone mineral density (BMD) changes in type 2 diabetes patients [8]. Furthermore, the serum TMAO level was significantly higher in postmenopausal women with hip fractures than in controls [9]. These findings strongly indicated that abnormally increased TMAO may contribute to bone loss in osteoporosis.

Bone loss during osteoporosis attributes to the imbalance of bone formation and bone resorption [10]. The potential of bone resorption is represented by osteoclast differentiation. Osteoclasts are derived from hematopoietic stem cells in the bone marrow [11]. After being stimulated by macrophage colony-stimulating factor (M-CSF), hematopoietic stem cells differentiate into bone marrow-derived macrophages (BMMs). BMMs then differentiate into preosteoclasts after being stimulated by RANKL. Preosteoclasts further fuse to form giant multinucleated mature osteoclasts after being stimulated for about 4 days. Mature osteoclasts can tightly attach to the bone surface by forming actin rings and lead to bone resorption [12]. In osteoclast differentiation, reactive oxygen species (ROS) production in BMMs is elevated after being stimulated by RANKL, followed by activation of NF-κB and MAPK signals [13,14]. Finally, the osteoclast differentiation-related downstream signals c-Fos and NFATc1 are stimulated to promote osteoclastogenesis [15]. However, the role and underlying mechanism of TMAO in regulating osteoclasts during osteoporosis are still unknown.

In the present study, we investigated whether TMAO would promote osteoclast differentiation in vitro and the NF-κB signaling pathway changes involved. Besides, we determined whether TMAO would induce bone loss in C57BL/6 mice and tested the in vivo osteoclast changes under TMAO. These findings provide exhaustive information for understanding the exact role and mechanisms of TMAO in osteoporosis.

## 2. Materials and Methods

### 2.1. Cell Culture

All experiments were approved by the Animal Ethical Committee of Shanghai Ninth People’s Hospital (SH9H-2022-A3-1). BMMs were isolated from the bone marrow of 4-week-old female C57BL/6 mice, which were purchased from Shanghai JieSJie Laboratory Animals Company. In brief, mice were sacrificed by cervical dislocation before being sterilized in 75% ethanol for 5 min. Then femurs and tibias were collected to flush out bone marrow cells. Afterward, the cells were cultured in α-MEM medium (HyClone, Logan, UT, USA) containing 10% (*v*/*v*) fetal bovine serum (Gibco, New York, NY, USA), 100 units/mL penicillin (HyClone, Logan, UT, USA), 100 μg/mL streptomycin (HyClone, Logan, UT, USA), and 30 ng/mL M-CSF (Peprotech, Cranbury, NJ, USA) at 5% CO_2_ and 37 °C [16,17]. 

### 2.2. Cell Viability Assay

Cell viability of BMMs under different concentrations of TMAO was evaluated by the cell counting kit (CCK-8, Dojindo Laboratories, Kumamoto, Japan). BMMs were seeded in 96-well plates at a density of 8 × 10^3^ cells per well. After treatment with TMAO for 24, 48, 72, and 120 h, the cells were incubated by 10% CCK-8 solution at 37 °C for 1 h. Then, the absorbance was measured at 450 nm using a microplate spectrophotometer (Tecan, M200pro, Mannedorf, Switzerland).

### 2.3. In Vitro Experiments

For osteoclast differentiation, BMMs were incubated with 30 ng/mL M-CSF and 100 ng/mL RANKL (Peprotech, Cranbury, NJ, USA). BMMs without any treatment were regarded as the control group. The other groups were treated with different doses of TMAO. Then, TRAP staining, F-actin staining, bone resorption assay, qRT-PCR, Western blot, and ROS detection were conducted. 

To determine whether the NF-κB signaling pathway was involved in TMAO-induced osteoclast differentiation, BMMs cells were pretreated with 10 μM of the NF-κB inhibitor, BAY 11-7082 (MCE, Shanghai, China) for 1 h [18]. In detail, BMMs were first incubated with 30 ng/mL M-CSF and 100 ng/mL RANKL, and then treated with 0 μM TMAO, 0 μM TMAO with BAY 11-7082, 400 μM TMAO, and 400 μM TMAO with BAY 11-7082, respectively.

To demonstrate whether ROS participated in the activation of NF-κB signals in the presence of TMAO, BMMs were pretreated with 30 mM of the ROS inhibitor, N-acetylcysteine (NAC, MCE, Shanghai, China) for 1 h [19]. In detail, BMMs were first incubated with 30 ng/mL M-CSF and 100 ng/mL RANKL, and then treated with 0 μM TMAO, 0 μM TMAO with NAC, 400 μM TMAO, and 400 μM TMAO with NAC, respectively.

### 2.4. Tartrate-Resistant Acid Phosphatase (TRAP) Staining

After TMAO treatment for 4 days, osteoclasts were fixed in 4% paraformaldehyde solution at 37 °C for 15 min and then stained with a TRAP staining kit (Sigma-Aldrich, Louis, MO, USA) according to the manufacturer’s instructions. After being incubated at 37 °C in the dark for 40 min, the number of TRAP-positive osteoclasts was observed by open-field microscopy (Olympus, IX71, Tokyo, Japan) and analyzed by ImageJ software (NIH, Bethesda, MD, USA).

### 2.5. F-Actin Ring Formation Assay

The F-actin ring is required for mature osteoclast formation and bone resorption [20]. To assess the formation of F-actin rings, the osteoclasts were fixed in 4% paraformaldehyde solution for 15 min and washed with PBS. After being treated with 0.3% Triton X-100, the osteoclasts were stained with rhodamine-conjugated phalloidin (Abcam, UK) for 60 min and then stained with 4′,6-diamidino-2-phenylindole (DAPI, Sigma-Aldrich, Louis, MO, USA) for 5 min. The F-actin rings were observed by a fluorescence microscope (Olympus, IX71, Japan) and analyzed by ImageJ software (NIH, Bethesda, MD, USA).

### 2.6. Bone Resorption Assay

BMMs were seeded on sterilized bovine bone slices and treated with different concentrations of TMAO. Two weeks later, the osteoclasts on the bone slices were removed by 0.25% EDTA-Trypsin. After dehydration and spray-gold treatment, the bone resorption area was observed under a scanning electron microscope (SEM, HITACHI, S4800, Tokyo, Japan) and analyzed by ImageJ software (NIH, Bethesda, MD, USA).

### 2.7. Quantitative Real-Time Polymerase Chain Reaction (qRT-PCR)

Total RNA was extracted using Trizol reagent (Invitrogen, Waltham, MA, USA), and 1000ng total RNA was reverse-transcribed by cDNA Synthesis SuperMix (Bimake, Houston, TX, USA). Real-time PCR was performed using SYBR Green qPCR Master Mix (Bimake, USA) on a real-time PCR platform (QuantStudio(TM) 6 Flex System, Waltham, MA, USA). All genes were normalized to GAPDH and calculated by the 2−ΔΔCt method. The primer sequences were as follows [16]: Calcr (Forward: 5′-CGGACTTTGACACAGCAGAA-3′, Reverse: 5′-AGCAGCAATCGACAAGGAGT-3′); Traf6 (Forward: 5′-AAACCACGAAGAGGTCATGG-3′, Reverse: 5′- GCGGGTAGAGACTTCACAGC-3′); Dcstamp (Forward: 5′-AAAACCCTTGGGCTGTTCTT-3′, Reverse: 5′-AATCATGGACGACTCCTTGG-3′); Acp5 (Forward: 5′-CACTCCCACCCTGAGATTTGT-3′, Reverse: 5′-CATCGTCTGCACGGTTCTG-3′); C-Fos (Forward: 5′-CCAGTCAAGAGCATCAGCAA-3′, Reverse: 5′-AAGTAGTGCAGCCCGGAGTA-3′); NFATc1 (Forward: 5′-CAGCTCCTGCTCCTCCTCC-3′, Reverse: 5′-CACATAACTGTAGTGTTCTTCCTCG-3′); GAPDH (Forward: 5′-AAGAGGGATGCTGCCCTTAC-3′, Reverse: 5′-CCAATACGGCCAAATCCGTTC-3′).

### 2.8. Western Blot

The total protein was extracted by a RIPA lysis buffer (Beyotime Biotechnology, Shanghai, China) containing 1 mM proteinase and phosphatase inhibitors. The concentration of total protein was determined by a BCA protein assay kit (Beyotime Biotechnology, China). Then, the protein extracts were subjected to SDS-PAGE and transferred to 0.22 μm PVDF membranes (Millipore, Bedford, MA, USA). The membranes were then blocked in 5% skim milk or 5% bovine serum albumin before being incubated in primary antibody solution overnight. The next day, the membranes were incubated in secondary antibody for 1 h in the dark and then detected by an infrared imaging system (Odyssey, Hastings, NE, USA).

The primary antibodies used were as follows: NFATc1 (Affinity Biosciences, DF6446, Jiangsu, China, 1:1000), C-Fos (Proteintech, 66590-1-Ig, Wuhan, China, 1:500), β-actin (Affinity Biosciences, T0022, Jiangsu, China, 1:5000), p-IKKα/β (Cell Signaling Technology, 2697, Danvers, MA, USA, 1:1000), IKKα (Cell Signaling Technology, 2682, Danvers, MA, USA, 1:1000), p-IKBα (Cell Signaling Technology, 2859, Danvers, MA, USA, 1:1000), IKBα (Cell Signaling Technology, 4812, Danvers, MA, USA, 1:1000), p-p65 (Cell Signaling Technology, 3033, Danvers, MA, USA, 1:1000), and p65 (Cell Signaling Technology, 8242, Danvers, MA, USA, 1:1000).

### 2.9. Intracellular ROS Assay

For ROS detection, the BMMs were stimulated with or without RANKL for 3 days and then probed with 10 μM DCFH-DA (Beyotime Biotechnology, Shanghai, China) dissolved in serum-free medium at 37°C for 30 min after being treated with different concentrations of TMAO. The fluorescence was measured at 488 nm excitation/525 nm emission by the laser confocal scanning microscope (LCSM, Leica, Wetzlar, Germany).

### 2.10. In Vivo Experiments

Twelve-week-old C57BL/6 mice were housed under a 12 h dark/light cycle at 23.1°C, 50–60% humidity, and allowed food and water ad libitum. Mice were randomly divided into two groups: the control and the TMAO group (*n* = 8 for each group). The mice in the two groups were allowed free access to sterile water or 1.5% TMAO solution for 8 weeks. The dosage of 1.5% TMAO was selected based on a previous report [21]. Humane euthanasia of mice was performed by cervical dislocation. During the experiment, the mice did not exhibit abnormal behavior. After cervical dislocation, bilateral femurs were collected for micro-CT, histology, and immunohistochemical analyses. The BMMs in the two groups were cultured and stimulated for qRT-PCR and Western blot analyses.

### 2.11. Micro-CT Analysis

Femur samples were scanned with a micro-CT 81 system (Scanco Medical AG, Bruettiselien, Switzerland) [22]. The parameters were set as follows: 70 kV (voltage), 114 μA (electric current), and 10 μm (resolution). After scanning, a 2–3 mm trabecular region of distal femur was selected as a volume of interest (VOI). Bone mineral density (BMD), bone volume (BV), trabecular bone volume fraction (BV/TV), trabecular number (Tb.N), trabecular thickness (Tb.Th), and trabecular spacing (Tb.Sp) were computed by Scanco Medical software (Scanco Medical AG, Bruettiselien, Switzerland).

### 2.12. HE, TRAP, and Immunohistochemical Staining

The femurs were embedded in paraffin after decalcification and then cut longitudinally into sections of 4–7 µm thickness. The femurs were stained with hematoxylin and eosin (HE) and a TRAP staining kit (Servicebio, Wuhan, China). Osteoclast number per bone surface (N. Oc/BS) and osteoclast surface per bone surface (Oc. S/BS) were quantified with Image-Pro Plus software (Media Cybernetics, Rockville, MD, USA).

For immunohistochemical analysis, the femur sections were incubated with anti-p65 antibody (Servicebio, Wuhan, China) at 4 °C overnight. Then, after being blocked by 5% BSA and 0.1% Triton X-100, the femurs were incubated with HRP-anti-rabbit IgG (1:200) at room temperature for 1 h. Digital images were captured by Olympus light microscopy (Olympus, Tokyo, Japan) and examined by ImageJ software (NIH, Bethesda, MD, USA).

### 2.13. RNA and Protein Extraction

After sacrificing, femurs and tibias were collected to flush out the bone marrow cells. BMMs were cultured with 30 ng/mL M-CSF and 100 ng/mL RANKL for 3 days. Then, total RNA and protein were extracted, qRT-PCR and Western blot were conducted. For qRT-PCR, the c-Fos and NFATc1 were detected. For Western blot, we detected the expression of c-Fos, NFATc1, and p65.

### 2.14. Statistical Analysis

All data of normal distribution were presented as means ± standard deviation (SD) and analyzed with GraphPad Prism 9.0 software (GraphPad Software Inc., La Jolla, CA, USA). The Student’s *t*-test was performed to compare the difference between the control group and the TMAO group in vivo. One-way analysis of variance (ANOVA) followed by a Tukey’s test was used to compare multigroup parametric data. Any *p* values < 0.05 was considered statistically significant. All experiments were conducted at least three times.

## 3. Results

### 3.1. Effects of TMAO on the Viability of BMMs

**Figure 1:**
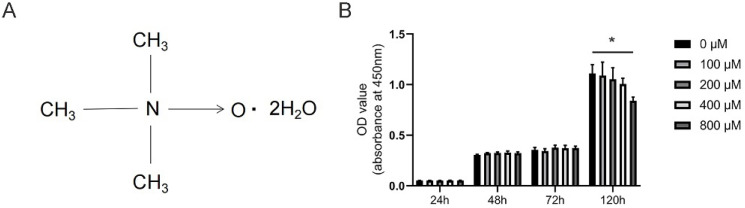
Effects of TMAO on the viability of BMMs. (**A**) Molecular structure of TMAO. (**B**) The cell viability of BMMs after treatment with different concentrations of TMAO for 24, 48, 72, and 120 h was evaluated by CCK-8. The cell viability of BMMs decreased significantly at 120 h with 800 μM TMAO treatment; * *p* < 0.05.

The chemical structure of TMAO is presented in Figure 1A. To determine the appropriate dosage of TMAO used in this study, CCK-8 assay was conducted. The number of BMMs decreased significantly at 120 h with 800 μM TMAO treatment (* *p* < 0.05) (Figure 1B). Therefore, the maximum concentration of TMAO used in the subsequent analysis was 400 μM.

### 3.2. TMAO Promoted Osteoclast Differentiation

**Figure 2:**
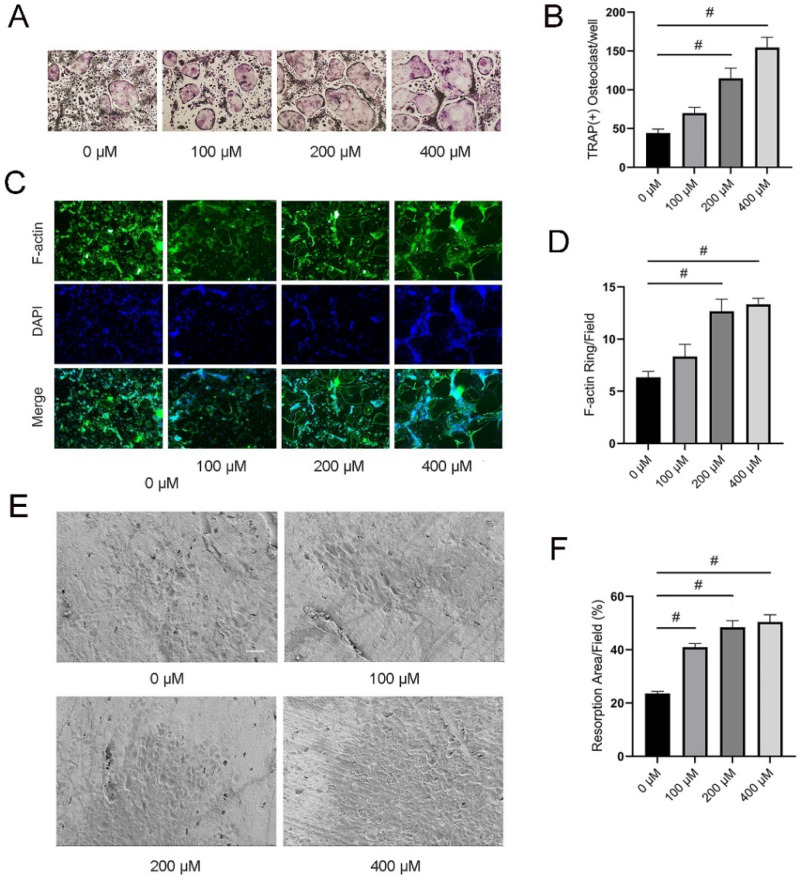
TMAO promoted osteoclast differentiation and resorption. (**A**,**B**) TMAO promoted TRAP-positive osteoclast formation in a dose-dependent manner. Scale bar = 20 µm. BMMs were cultured in α-MEM containing 30 ng/mL M-CSF and 100 ng/mL RANKL under different concentrations of TMAO for 4 days. Osteoclasts were stained by a TRAP staining kit. TRAP-positive osteoclasts were observed and counted by open-field microscopy. (**C**,**D**) TMAO increased F-actin ring formation in a dose-dependent manner. Scale bar = 200 µm. BMMs were cultured in α-MEM containing 30 ng/mL M-CSF and 100 ng/mL RANKL under different concentrations of TMAO for 4 days. The cells were stained with rhodamine-conjugated phalloidin for 60 min and then stained with 4′,6-diamidino-2-phenylindole for 5 min. Then the F-actin rings were observed by a fluorescence microscope. (**E**,**F**) TMAO increased bone resorption area in a dose-dependent manner. Scale bar = 50 µm. BMMs were seeded on the sterilized bovine bone slices and treated with different concentrations of TMAO for two weeks. The bone resorption area was observed under a scanning electron microscope. ^#^ *p* < 0.01.

TRAP-positive osteoclasts were significantly increased under 200 and 400 μM TMAO treatment (^#^ *p* < 0.01; Figure 2A,B). Moreover, the average number of F-actin rings significantly increased following treatment with 200 and 400 μM TMAO (^#^ *p* < 0.01; Figure 2C,D). Furthermore, bone resorption by osteoclasts was enhanced with the increase in TMAO (Figure 2E). Quantitative analysis of resorption area showed that the percentage of resorption area per field were significantly increased with TMAO in a dose-dependent manner (^#^ *p* < 0.01; Figure 2F). These results demonstrated that TMAO could promote osteoclast differentiation.

### 3.3. TMAO Enhanced Osteoclast Gene Expression and NF-κB Signaling Pathway

**Figure 3:**
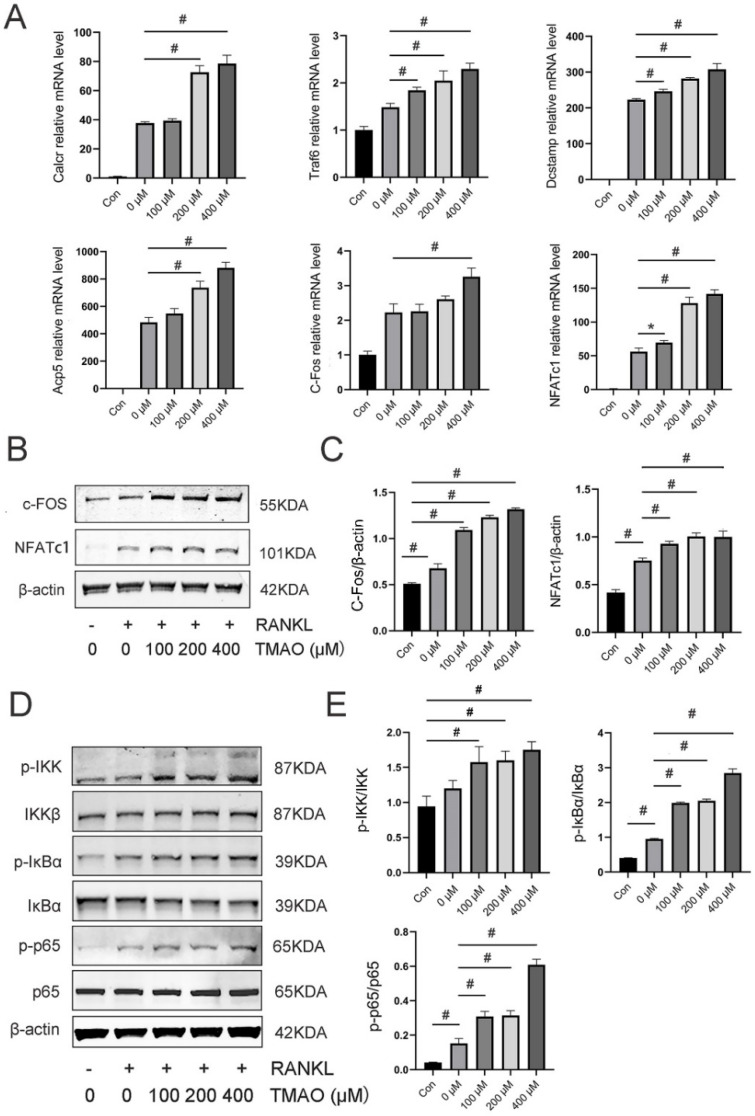
TMAO enhanced osteoclast gene expression and the NF-κB signaling pathway. (**A**) TMAO enhanced osteoclast gene expression (*n* = 3 per group) as examined by real-time PCR. (**B**,**C**) TMAO enhanced c-Fos and NFATc1 protein expression as evaluated by Western blot (*n* = 3 per group). (**D**,**E**) The protein expression levels of *p*-IKK, IKK, p-IκBα, IκBα, p-p65, and p65 were examined by Western blot after TMAO treatment. TMAO increased RANKL-stimulated phosphorylation of IKK, IκBα, and p65 (*n* = 3 per group). * *p* < 0.05, ^#^ *p* < 0.01.

Next, we detected the crucial genes during osteoclast differentiation under TMAO. The expression of Calcr, Traf6, Dcstamp, Acp5, C-Fos, and NFATc1 was significantly increased following TMAO treatment (* *p* < 0.05; ^#^ *p* < 0.01) (Figure 3A). Moreover, protein expression of the downstream osteoclast-related transcription factors C-Fos and NFATc1 was also enhanced under TMAO (Figure 3B,C). These results further suggested that TMAO could promote osteoclast differentiation in vitro.

The NF-κB signaling pathway is of great importance in osteoclast differentiation. Phosphorylation of IKKα/β, IκBα, and p65 relative to total IKKβ, IκBα, and p65 was significantly increased in a dose-dependent manner with the treatment of TMAO (Figure 3D,E). These findings demonstrated that the NF-κB signaling pathway was activated when TMAO induced osteoclast differentiation.

### 3.4. Inhibition of the NF-κB Signaling Pathway with BAY 11-7082 Reversed the Effect of TMAO on Osteoclast Differentiation

**Figure 4:**
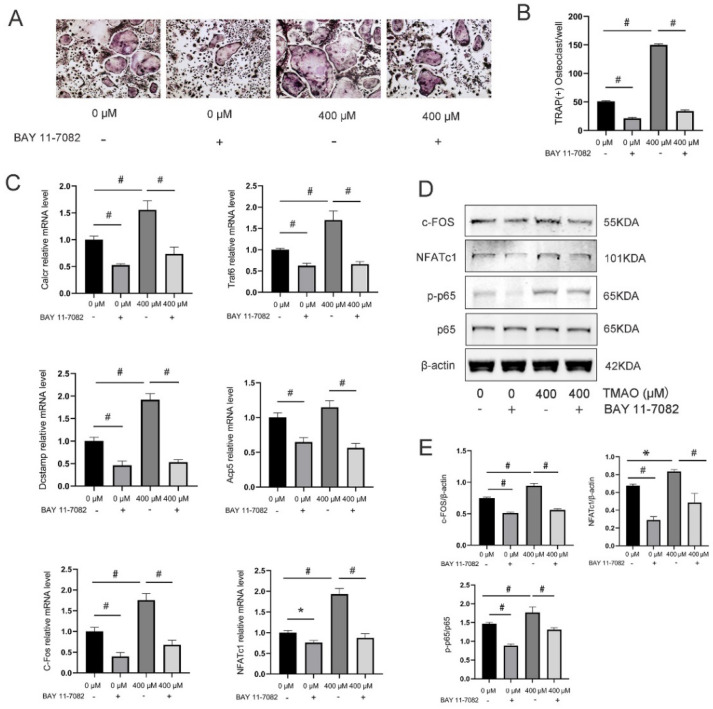
Inhibition of the NF-κB signaling pathway with BAY 11-7082 reversed the effect of TMAO on osteoclast differentiation. (**A**,**B**) BMMs were pretreated with 10 μM BAY 11-7082 for 1 h before being cultured with or without 400 µM TMAO. The number of TRAP-positive osteoclasts increased under 400 μM TMAO and decreased with BAY 11-7082 pretreatment with or without TMAO. Scale bar = 20 µm. (**C**) The expression of osteoclast-specific genes was up-regulated under 400 μM TMAO and suppressed by BAY 11-7082 with or without TMAO treatment evaluated by real-time PCR (*n* = 3 per group). (**D**,**E**) The protein expression levels of c-Fos, NFATc1, and the phosphorylation of p65 were promoted under 400 μM TMAO and suppressed by BAY 11-7082 with or without TMAO treatment as examined by Western blot (*n* = 3 per group). Con: blank control group without RANKL and TMAO; * *p* < 0.05, ^#^ *p* < 0.01.

BAY 11-7082 is a commonly used NF-κB inhibitor that can block the phosphorylation of IκBα and NF-κB p65 [23]. We proved that TMAO could induce osteoclast differentiation and NF-κB activation. To further investigate the key role of the NF-κB signaling pathway in TMAO-promoted osteoclast differentiation, we added BAY 11-7082 to determine whether osteoclast differentiation with the presence of TMAO could be suppressed. First, the number of TRAP-positive osteoclasts increased under 400 μM TMAO and decreased with BAY 11-7082 pretreatment with or without TMAO (^#^ *p* < 0.01; Figure 4A,B). Second, the increased expression of Calcr, Traf6, Dcstamp, Acp5, C-Fos, and NFATc1 under TMAO all up-regulated under 400 μM TMAO and down-regulated with BAY 11-7082 pretreatment (* *p* < 0.05, ^#^ *p* < 0.01; Figure 4C). Third, the protein expression of C-Fos and NFATc1 was also increased under 400 μM TMAO treatment and decreased with BAY 11-7082 treatment (Figure 4D,E). Finally, and as expected, the expression of *p*-p65/p65 decreased when using BAY 11-7082 to inhibit the NF-κB signaling pathway (Figure 4D,E). These results implied that the promotional effect of TMAO on osteoclast differentiation was regulated by the NF-κB signaling pathway.

### 3.5. TMAO Increased ROS Levels during Osteoclast Differentiation

**Figure 5:**
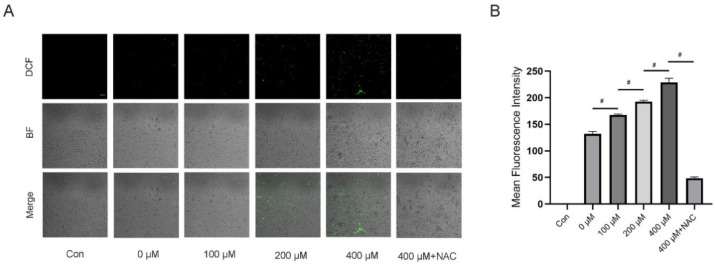
TMAO increased ROS levels in BMMs during osteoclast differentiation. (**A**) Representative confocal images of intracellular ROS generation. Scale bar = 100 µm. (**B**) BMMs were stimulated with or without RANKL for 3 days and then probed with 10 μM DCFH-DA for 30 min after being treated with different concentrations of TMAO. TMAO treatment led to increased fluorescence intensity of ROS in BMMs in a dose-dependent manner, and NAC treatment decreased TMAO-induced ROS generation (*n* = 3 per group). ^#^ *p* < 0.01.

Several previous studies have indicated that ROS play a critical role as intracellular messenger molecules to activate the downstream signaling pathways in the initiation of osteoclast differentiation [14,16,17]. Moreover, TMAO has been proven to be associated with oxidative stress and overproduction of ROS during inflammatory state. To investigate whether ROS was involved in TMAO-promoted osteoclast differentiation, we determined the ROS levels in BMMs under TMAO. The results showed that TMAO treatment led to increased fluorescence intensity of ROS in BMMs in a dose-dependent manner (^#^ *p* < 0.01; Figure 5A,B). However, ROS generation decreased when using NAC to neutralize intracellular ROS (^#^ *p* < 0.01; Figure 5A,B). These findings suggested that intracellular ROS was activated in TMAO-promoted osteoclast differentiation.

### 3.6. Inhibition of ROS by NAC Suppressed TMAO-Induced NF-κB Activation

**Figure 6:**
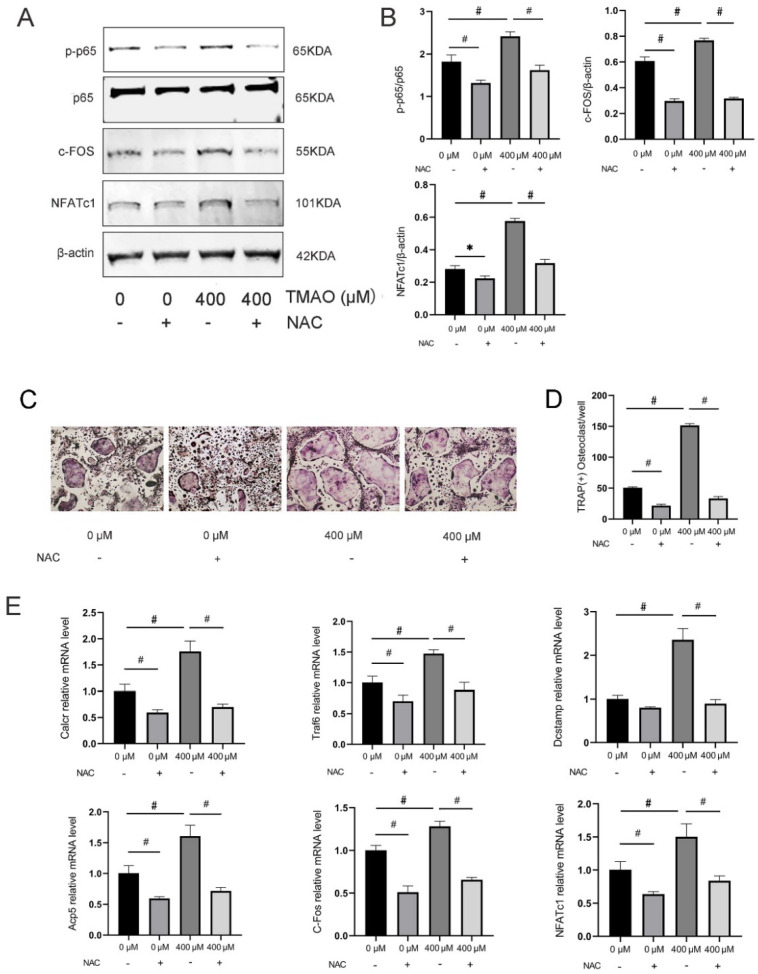
Inhibition of ROS by NAC suppressed TMAO-induced NF-κB activation. (**A**,**B**) The phosphorylation of p65, and the protein expression levels of c-Fos, NFATc1 were promoted under 400 μM TMAO and suppressed by NAC with or without TMAO treatment as examined by Western blot (*n* = 3 per group). (**C**,**D**) BMMs were pretreated with 30 mM NAC for 1 h before being cultured with or without 400 µM TMAO. The numbers of TRAP-positive osteoclasts increased with 400 μM TMAO and decreased with NAC pretreatment with or without TMAO. Scale bar = 20 µm. (**E**) The expression of the osteoclast-specific genes was promoted under 400 μM TMAO and suppressed by NAC with or without TMAO treatment as evaluated by real-time PCR (*n* = 3 per group). * *p* < 0.05, ^#^ *p* < 0.01.

So far, we have proven that TMAO could induce osteoclast differentiation by activating the NF-κB signaling pathway. We also found that TMAO could increase ROS level in osteoclast differentiation. Given that TMAO and ROS both activated the NF-κB signaling pathway and induced osteoclast differentiation, ROS might play a crucial role in linking TMAO and the NF-κB signaling pathway in osteoclast differentiation. Next, to determine whether TMAO-promoted osteoclast differentiation by the NF-κB signaling pathway is ROS-dependent, we added NAC to determine whether NF-κB activation and osteoclast differentiation with the presence of TMAO could be suppressed. The results showed that NAC pretreatment down-regulated the critical NF-κB signaling p-p65/p65 expression in BMMs treated with 400 μM TMAO (Figure 6A,B), which suggested that ROS was activated before the NF-κB signaling pathway in TMAO-promoted osteoclast differentiation. We also found that TRAP-positive osteoclasts increased under 400 μM TMAO and decreased under NAC with or without TMAO (^#^ *p* < 0.01; Figure 6C,D). Moreover, the osteoclast-differentiation-related genes Calcr, Traf6, Dcstamp, Acp5, C-Fos, and NFATc1 decreased when treating the BMMs with NAC (^#^ *p* < 0.01; Figure 6E). The protein expression of C-Fos and NFATc1 was also promoted under 400 μM TMAO and decreased with NAC in TMAO-treated osteoclasts. Taken together, these results demonstrated that TMAO could promote in vitro osteoclast differentiation through the ROS-dependent NF-κB signaling pathway.

### 3.7. TMAO Induced Bone Loss in Mice

**Figure 7:**
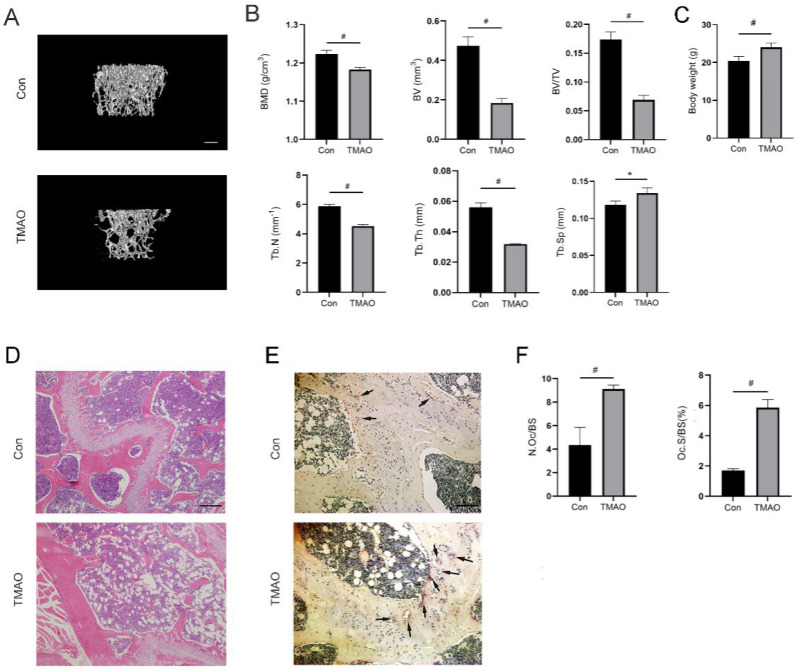
TMAO induced bone loss in mice. (**A**) Representative micro-CT reconstruction images of femur from mice of control and TMAO groups. Scale bar = 400 µm. (**B**) Bone parameters of BMD, BV, BV/TV, Tb.N, and Tb.Th in the TMAO group were significantly lower than in the control group, and Tb.Sp in the TMAO group was significantly higher than in the control group (*n* = 4 per group). (**C**) Body weight of mice in the TMAO group was significantly higher than in the control group (*n* = 6 per group). (**D**) Representative images of HE staining of femur from mice of control and TMAO groups. Scale bar = 5 μm. (**E**) Representative images of TRAP staining of femur from mice of control and TMAO groups. TRAP-positive osteoclasts are marked by arrows. Scale bar = 5 μm. (**F**) TMAO significantly increased osteoclast number/bone surface area (N.Oc/BS) and osteoclast area/bone surface area (Oc.S/BS) observed by TRAP staining (*n* = 3 per group). * *p* < 0.05, ^#^ *p* < 0.01.

In order to test whether TMAO would induce bone loss in vivo, TMAO was given to mice in drinking water with the concentration of 1.5% for 8 weeks. The qualitative images showed an obvious bone loss in the TMAO group compared to the control group (Figure 7A). Quantification data revealed that BMD, BV, and BV/TV in the TMAO group were significantly lower than in the control group, as were Tb.N and Tb.Th (^#^ *p* < 0.01; Figure 7B), which suggested that TMAO could reduce trabecular bone mass in mice. As for body weight, mice in the TMAO group had significantly higher body weight than in the control group (Figure 7C). In addition, HE staining showed that the number and thickness of trabeculae were reduced in the TMAO group (Figure 7D). TRAP staining and quantification showed that more TRAP-positive osteoclasts existed in the TMAO group than in the control group (^#^ *p* < 0.01; Figure 7E,F). These results suggested that TMAO could induce bone loss in mice, which was related to increased osteoclasts formed in vivo.

### 3.8. TMAO Increased the Expression of Osteoclast Genes and NF-κB Signaling in Mice

**Figure 8:**
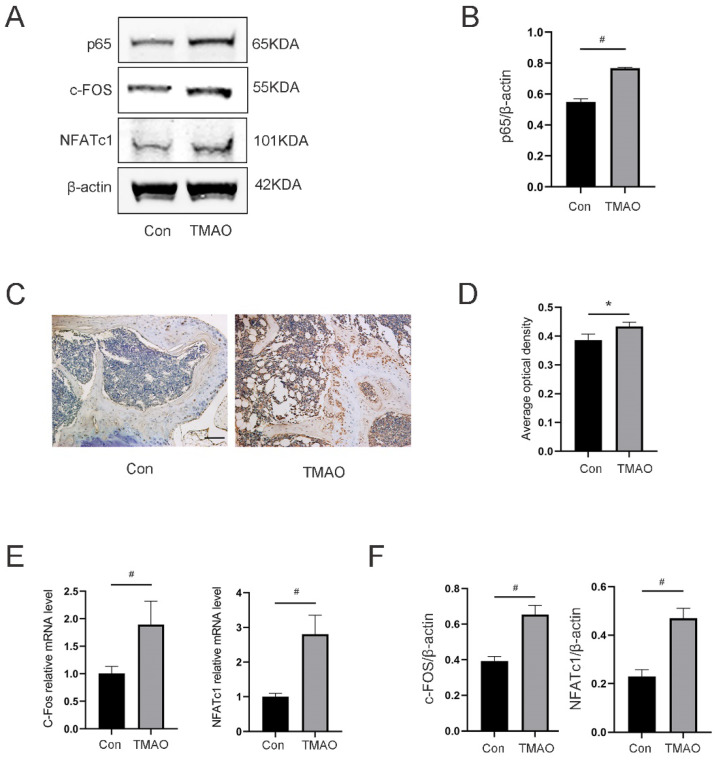
TMAO increased the expression of osteoclast genes and NF-κB signaling in mice. (**A**) Representative Western blot bands of NF-κB p65, c-Fos and NFATc1 relative to β-actin. (**B**) The expression of NF-κB p65 in the TMAO group was significantly increased compared to the control group (*n* = 3 per group). (**C**) Representative femur section images of immunohistochemical staining of p65 from mice of control and TMAO groups. Scale = 5 μm. (**D**) TMAO significantly promoted average optical density (AOD) of p65. (**E**) TMAO significantly promoted C-Fos and NFATc1 gene expression in bone marrow examined by real-time PCR in vivo (*n* = 3 per group). (**F**) TMAO significantly promoted C-Fos and NFATc1 protein expression in bone marrow examined by Western blot in vivo (*n* = 3 per group). * *p* < 0.05, ^#^ *p* < 0.01.

As we had witnessed TMAO could promote osteoclast differentiation by activating the ROS-dependent NF-κB signaling pathway in vitro and bone loss in mice, we further tested whether NF-κB signaling would be activated in vivo during TMAO-induced bone loss in mice. Western blot showed that the expression of total NF-κB p65 in the TMAO group was significantly increased compared to the control group (Figure 8A,B). Besides, the immunohistochemical analysis showed increased NF-κB p65 expression in the TMAO group (Figure 8C,D). The gene and protein expression of C-Fos and NFATc1 was increased in BMMs from TMAO-treated mice (Figure 8E,F). Taken together, these data manifested that TMAO could induce bone loss in mice, which was related to activation of the NF-κB signaling pathway.

## 4. Discussion

Gut microbiota composition and its products play important roles in primary and secondary osteoporosis [24]. TMAO is a GM product that has been shown to be associated with high risk of hip fractures in postmenopausal women, suggesting that increased TMAO may contribute to bone loss and fracture in postmenopausal women [8]. However, limited information is known about its role in bone loss during osteoporosis. In the present study, for the first time, we demonstrated that TMAO could promote osteoclast differentiation in vitro, which was achieved by activating the ROS-dependent NF-κB signaling pathway. Besides, we found that TMAO could induce bone loss in mice, which was the result of the in vivo increased osteoclasts caused by enhanced expression of the NF-κB signaling pathway. These findings would be of great meaning to the understanding of the mechanisms by which TMAO contributes to bone loss in osteoporosis.

A recent study showed that 200 μM TMAO could significantly decrease the BMSCs viability [18]. However, 600 μM TMAO did not show any negative effect on cell viability of vascular smooth muscle cells [25]. In this study, the concentration threshold of TMAO was set to 400 μM in the in vitro studies according to the cell viability test. Therefore, different TMAO concentrations were selected in these studies. These inconsistent dosages suggested that TMAO would have different degrees of toxic effects on different cell lines. On the other hand, we gave the mice 1.5% TMAO by oral administration in drinking water, which was similar to a previous TMAO animal study [21]. Besides, 1.5% TMAO in drinking water is equivalent to the concentration of 150 mM in plasma, which was close to the physiological concentration of TMAO in plasma (200 mM) [26]. From this point of view, our animal experiment simulated the clinical situation to some extent. Therefore, the concentrations we set in the current study were appropriate.

TMAO was witnessed to be a risk factor for various chronic inflammatory diseases. As previously reported, TMAO could induce CVDs by directly stimulating inflammation in vascular smooth muscle cells (VSMCs) [25]. By inducing atherosclerosis and thrombosis vascular fibrosis, TMAO could also affect renal function and finally lead to chronic kidney disease (CKD) [27]. Epidemiological studies have provided evidence that patients with CVD are at higher risk for osteoporosis [28,29]. Moreover, atherosclerosis has the common pathophysiological mechanisms to osteoporosis, including oxidative stress and inflammation [30]. In the current study, we showed that TMAO could induce bone loss in mice. Given that people suffering from cardiovascular disease, chronic kidney disease, and diabetes tend to face a higher risk of osteoporosis, modulating serum TMAO may alleviate osteoporosis in these individuals. The current study may extend the possibility of using TMAO as a therapeutic target for osteoporosis, especially in patients with basic diseases.

The NF-κB signaling pathway regulates many cellular processes including inflammation, immune response, and apoptosis [31]. A recent study validated that TMAO activated NF-κB signals during vascular calcification in CKD rats. Besides, inhibition of NF-κB signals attenuated TMAO-induced vascular calcification [25]. Another study found that TMAO induced vascular inflammation by activating NF-κB signals in atherogenesis [32]. It has been well established that the NF-κB signaling pathway is essential for the stimulation of osteoclast differentiation [12]. However, the regulating effect of the NF-κB signaling pathway on TMAO-induced osteoclast differentiation is still unknown. In the current study, we validated that abnormally increased TMAO could promote osteoclast differentiation by activating the NF-κB signals. Therefore, intervention of NF-κB signals might be promising to attenuate TMAO-induced bone loss.

Oxidative stress is triggered by the constant production of ROS generated by inflammation and mitochondrial dysfunction [33]. Studies have reported that ferric ion, cerium ion, and several compounds, such as NOX4, duoxa1, homocysteine, and glucocorticoid, could promote osteoclast differentiation by inducing ROS accumulation [34,35,36,37,38,39]. Accumulating evidence proves that TMAO could induce oxidative stress and significantly enhance intracellular ROS level in various vascular pathological processes, including atherosclerosis, vascular inflammation, and vascular aging [40,41,42,43]. In addition, ROS is an upstream signaling molecule to activate NF-κB signals, which is critical for osteoclast differentiation [44]. In the present study, we found that TMAO could also induce osteoclast differentiation via the stimulation of ROS. Therefore, TMAO-induced bone loss in osteoporosis could be caused by an inflammatory process triggered by oxidative stress.

As for osteoclast differentiation, we did not investigate the direct mechanism of TMAO-promoted ROS overproduction in this study. However, in a previous study on TMAO-induced vascular inflammation, the SIRT3-SOD2 linked pathway was found to mediate mitochondrial ROS (mtROS) generation [42]. The SIRT3-SOD2 linked pathway is critical for regulating ROS production. To maintain ROS homeostasis, SIRT3 activates SOD2 via deacetylation and converts superoxide radicals to harmless oxygen or hydrogen peroxide [45]. However, whether the SIRT3-SOD2 linked pathway or any other signal is involved in the TMAO-induced ROS production in osteoclast differentiation needs to be further investigated.

TMAO is produced by GM-mediated metabolism of choline, betaine, and L-carnitine. Several bacteria of GM, including Gammaproteobacteria, Betaproteobacteria, Firmicutes, and Actinobacteria, can produce TMAO [5]. In previous studies, GM was established to be closely associated with primary osteoporosis [46,47]. GM is also essential for secondary osteoporosis [48,49]. Furthermore, GM transplanted from children alleviated bone loss in ovariectomy (OVX)-induced osteoporotic mice [50]. by improving gut microbiome composition. These studies imply that it may be possible to treat osteoporosis with the regulation of GM composition. In this study, we found that increased TMAO could contribute to bone loss in mice. Hence, modulating serum TMAO level by regulating gut microbiota or altering dietary structure would be a promising therapeutic method to alleviate osteoporosis. The current study extends the possibility of TMAO as a therapeutic target for osteoporosis, especially in patients with cardiovascular disease, chronic kidney disease, and diabetes.

A limitation of this study was that NF-κB and ROS inhibitors were not added in the in vivo experiment. We have demonstrated that TMAO can induce osteoclast differentiation via activating the ROS-dependent NF-κB signaling pathway in vitro. However, inhibitor experiments were not performed to confirm TMAO-induced bone loss in mice through the same pathway. The reason is that oxidative stress and the NF-κB signaling pathway are related to many other pathological processes besides osteoporosis. Furthermore, we primarily investigated the effects of TMAO on osteoclast differentiation instead of osteogenic differentiation. Osteoporosis is mainly caused by hyperactivity of osteoclasts. On the other hand, osteogenic differentiation also plays a critical role in the development of osteoporosis. Therefore, although we have indicated that TMAO leads to bone loss via inducing osteoclasts differentiation and NF-κB activation, the effect of TMAO on osteogenic differentiation in vivo still needs further investigation. Despite these limitations, our study demonstrated that TMAO induced osteoclast differentiation and bone loss, which were related to the ROS-dependent NF-κB signaling pathway. 

## 5. Conclusions

To our knowledge, this is the first study to clarify that TMAO directly promotes osteoclast differentiation by activating the ROS-dependent NF-κB signaling pathway. Moreover, TMAO could induce bone loss in mice by enhancing the osteoclasts. Overall, the findings of the current study provide exhaustive information to comprehend the exact mechanisms of TMAO in osteoporosis.

## Data Availability

Data are contained within this article.

## Abbreviations

TMAO: trimethylamine-N-oxide; GM: gut microbiota; NAC: N-acetylcysteine; M-CSF: macrophage colony-stimulating factor; RANKL: nuclear factor-kappaB ligand; BMD: bone mineral density; BMMs: bone-marrow-derived macrophages; ROS: reactive oxygen species.
